# A screening for antimicrobial activities of Caribbean herbal remedies

**DOI:** 10.1186/1472-6882-13-126

**Published:** 2013-06-04

**Authors:** Claribel Luciano-Montalvo, Isabelle Boulogne, Jannette Gavillán-Suárez

**Affiliations:** 1 Institute of Interdisciplinary Research, University of Puerto Rico, Cayey, #205 Antonio R. Barceló Ave, Cayey, PR 00736, Puerto Rico; 2 University of French Antilles and Guyana, UFR Faculty of Science, TRAMIL, F-97157, Pointe à Pitre, Guadeloupe Cedex, Guadeloupe; 3 Department of Chemistry, University of Puerto Rico, Cayey, #205 Antonio R. Barceló Ave, Cayey, PR 00736, Puerto Rico; 4 Present address: San Juan Bautista Medical School, Department of Microbiology, Caguas 00727, Puerto Rico

**Keywords:** Ethnopharmacology, TRAMIL, Antibacterial, *Syzygium jambos*, *Pityrogramma calomelanos*, *Tapeinochilus ananassae*, *Gossypium barbadense*

## Abstract

**Background:**

The TRAMIL program aims to understand, validate and expand health practices based on the use of medicinal plants in the Caribbean, which is a “biodiversity hotspot” due to high species endemism, intense development pressure and habitat loss. The antibacterial activity was examined for thirteen plant species from several genera that were identified as a result of TRAMIL ethnopharmacological surveys or were reported in ethnobotanical accounts from Puerto Rico. The aim of this study was to validate the traditional use of these plant species for the treatment of bacterial infections, such as conjunctivitis, fever, otitis media and furuncles.

**Methods:**

An agar disc diffusion assay was used to examine five bacterial strains that are associated with the reported infections, including *Staphylococcus saprophyticus* (ATCC 15305), *S. aureus* (ATCC 6341)*, Escherichia coli* (ATCC 4157), *Haemophilus influenzae* (ATCC 8142), *Pseudomonas aeruginosa* (ATCC 7700) and *Proteus vulgaris* (ATCC 6896), as well as the fungus *Candida albicans* (ATCC 752)*.* The minimum inhibitory concentration (MIC) and minimum bactericidal concentration (MBC) values were determined for each of the extracts that showed inhibitory activity.

**Results:**

The decoctions of *Pityrogramma calomelanos*, *Tapeinochilus ananassae*, and *Syzygium jambos,* as well as the juice of *Gossypium barbadense,* showed > 20% growth inhibition against several bacteria relative to the positive control, which was the antibiotic Streptomycin. Extracts with the best antimicrobial activities were *S. jambos* that showed MIC = 31 μg/mL and MBC = 1.0 mg/mL against *P. vulgaris* and *T. ananassae* that showed MIC = 15 μg/mL against *S. aureus*.

**Conclusion:**

This report confirms the traditional use of *P. calomelanos* for the treatment of kidney infections that are associated with stones, as well as the antimicrobial and bactericidal effects of *T. ananassae* against *P. vulgaris* and *S. saprophyticus* and the effects of *S. jambos* against *S. aureus* and *S. saprophyticus*.

## Background

The plant-based formulations are a viable option that could be useful in reducing the side effects associated with conventional antibiotic treatment [1]. Plant-based formulations would also increase the number of compounds that are used to limit infections [2] and might function synergistically with current therapies [3]. Many reports have identified anti-bacterial properties of plants, but these studies have primarily been conducted in Asia [4]. The Caribbean is one of the world’s centers of biodiversity and endemism identified as a tropical mountainous “biodiversity hotspot” because “high species endemism combines with proportionally extensive habitat loss” [5]. Information regarding the use of herbal remedies in the Caribbean was obtained during TRAMIL (Traditional Medicines in the Islands) surveys. TRAMIL, which is a program of applied research focusing on popular medicine, was initiated in 1982 to understand, validate and expand primary health care practices based on the use of medicinal plants in the Caribbean. The TRAMIL program has a unique methodology that consists of ethnopharmacological surveys and bibliographic and experimental research. The TRAMIL program works to validate plant species, plant organs and methods of preparation that are recorded during TRAMIL surveys and that are used frequently, 20% or more, for a particular ailment but are not documented in the scientific literature. The scientific validation of traditional health practices is based on criteria of safety and efficacy [6,7]. Moreover, TRAMIL organizes outreach activities (TRADIF) that are aimed at disseminating the results obtained from the efficacy and toxicity studies. Thus, the objectives and experience of TRAMIL in traditional Caribbean knowledge and the sustainable utilization of plants appear to be the best starting point to find interesting plants with antimicrobial potential.

In addition, plant species described in ethnobotanical accounts from Puerto Rico that are used to treat infections and as adjuvants for diabetes were included in this study [8,9]. There is evidence that diabetic patients suffer from high bacteremia levels due to high blood sugar levels that weaken the patient’s immune system defenses [10] and increase the body’s vulnerability to bacterial infections [11,12]. Consequently, the aim of this study was to validate the antimicrobial activity of herbal remedies that are used in the Caribbean to treat infections such as conjunctivitis, otitis media, kidney infections, and furuncles.

## Methods

### Plant collection and identification

All of the plants were collected in Puerto Rico. The specimens were numbered and deposited at the George Proctor Herbarium (SJ) in Puerto Rico. José Sustache, Botanist and Head of Puerto Rico Department of Natural and Environmental Resources Herbarium, classified the botanical species. The plant species that have been identified in the TRAMIL program and their traditional uses are the focus of the current study and are shown in Table 1.

**Table 1:** Plant species recorded in ethnopharmacological/ethnobotanical surveys in the Caribbean that were examined for their antimicrobial and antifungal activities

**Scientific name/(voucher specimen)**	**Country/Traditional use**^**a**^	**Local name**	**Plant parts used/Preparation form**	**Extracts yield (% w/w)**	**Concentration of extracts (mg/mL)**	**Concentration of extracts/disc (μg/μL)**^**b**^		
						**Disc 1**	**Disc 2**	**Disc 3**
Cucurbitaceae *Cucurbita moschata* Duchesne (019344)	Dominican Republic/skin burns	*auyama*	leaves/juice	2.5	50.0	50.0	25.0	12.5
Plantaginaceae *Plantago major* L. (019342)	Nicaragua/kidney infections	*llantén*	leaves/decoction	1.4	28.7	28.7	14.3	7.2
Labiatae *Plectranthus amboinicus* (Lour.) Spreng. (019352)	Dominican Republic/otitis	*orégano poleo*	leaves/juice	1.1	21.3	21.3	10.7	5.3
Scrophulariaceae *Capraria biflora* L. (019353)	Guadalupe, Marie-Galante, Martinique, Tobago/conjunctivitis	*Té péyi, paye, titi,*	leaves/decoction	5.6	112.6	112.6	56.3	28.1
Malvaceae *Abelmochus esculentus* (L.) Moench (019662)	Dominican Republic/eye infections	*molondrón*	fruit/juice	0.6	12.6	12.6	6.3	3.2
Malvaceae *Gossypium barbadense* L. (019366)	Haiti/otitis media	*koton*	leaves/decoction unripe fruit/juice	5.5 1.4	110.5 27.9	110.5 27.9	55.3 14.0	27.6 7.0
Malvaceae *Hibiscus rosa-sinensis* L. (019535)	Haiti/conjunctivitis	*choublak*	flower/juice	0.9	17.9	17.9	9.0	4.5
Pteridaceae *Pityrogramma calomelanos* (L.) Link (019372)	Tobago/kidney stones or urolithiasis	*stamp fern, egyptian secret*	leaves/decoction	7.5	150.0	150.0	75.0	37.5
Aristolochiaceae *Aristolochia trilobata* L. (019609)	fever	*bejuco estrella*	leaves/decoction	8.8	176.5	176.5	88.3	44.0
Commelinaceae *Tradescantia spathacea* Sw.^c^ (019664)	Puerto Rico/psoriasis	*sanguinaria*	leaves/decoction	2.8	56.0	56.0	28.0	14.0
Zingiberaceae *Costus specious* (J. König) Sm.^c,d^ (019660)	Puerto Rico/nephritis	*caña amarga, insulina*	leaves/decoction	2.4	47.3	47.3	23.7	11.8
Zingiberaceae *Tapeinochilus ananassae* K. Schum.^d^ (019553)	Puerto Rico	*insulina*	leaves/decoction	3.1	61.5	61.5	30.5	15.3
Myrtaceae *Syzygium jambos* (L.) Alston^d^ (019663)	Puerto Rico	*Pomarrosa del río*	leaves/decoction	2.1	41.8	41.8	20.9	10.5

a) Reported in 20% or more of the interviews for a given ailment.

b) Extracts concentration/disc: disc 1 - μg/μL in the re-dissolved lyophilized extract at the final concentration, discs 2 and 3 - μg/μL in 50% and 25% dilutions of the re-dissolved extract, respectively.

c) As reported in the ethnobotanical literature in Puerto Rico to exhibit antimicrobial properties.

d) As reported in ethnopharmacological survey in Puerto Rico as an adjuvant for diabetes.

### Plant material and preparation of decoctions, juices and aqueous extracts

Decoctions were prepared by boiling 30 g of plant material in 100 mL of distilled water. After concentration to 15 mL, to lyophilize 3 × 5 mL replicates, the decoctions were filtered through cheesecloth, and freeze-dried using a Freezone 4.5 lyophilizer. Plant juices (3 × 5 mL replicates) were lyophilized after the plant part was crushed in a mortar and filtered through cheesecloth. The solids obtained after freeze-drying were re-dissolved in autoclaved water to known concentrations for antibacterial determination. The concentrations of the lyophilized extracts in mg/mL were calculated as the average of the total solids obtained per mL of lyophilized decoction or plant juice. The plant part, preparation form, concentration of the re-dissolved extracts and the extraction yields are reported in Table 1.

### Disc preparation

Whatman chromatography no 2 filter paper was used to prepare 6.3 mm paper discs that were incubated with 20 μL aliquots of the re-dissolved extracts from lyophilized decoctions or juices at their final concentration and at 25% and 50% dilutions, and air-dried for 2 hours, as described previously [13]. The positive controls were Streptomycin (5 mg/mL) and Nystatin (50 mg/L) for the antibacterial and antifungal assays, respectively. Distilled water was used as a negative control. The concentration of the extracts in the paper discs is reported in Table 1.

### Bacterial selection and culture

The Caribbean Herbal Pharmacopeia [6], which is published by the TRAMIL program, established the experimental models that are required to validate antimicrobial activity. In this case, the antimicrobial activity had to be confirmed for at least one case. For agents associated with bacterial conjunctivitis, TRAMIL requires *in vitro* susceptibility studies of *Haemophilus influenzae*, *Streptococcus pneumonia* or *Staphylococcus aureus*. For otitis media, TRAMIL recommends the analysis of the effects of herbal remedies against the Gram-negative bacteria *Escherichia coli*, *Pseudomonas aeruginosa* or *Proteus vulgaris*; the Gram-positive bacterium *S. aureus*; and the fungus *Candida albicans*. *E. coli* is the primary causal agent of urinary tract infections (UTIs) and accounts for > 75% of community-acquired UTIs in all age groups, whereas *Staphylococcus saprophyticus* accounts for 10% of total UTIs [14]. Infections due to the presence of kidney stones are more commonly caused by urease-producing bacteria, such as *S. aureus*, *Pseudomonas* and *Proteus*. Finally, in the case of furuncle, which is an infection of the hair follicle, TRAMIL requires validation of antimicrobial activity against S*. aureus*.

The test organisms included the Gram-positive bacteria *Staphylococcus saprophyticus* (ATCC 15305) and *Staphylococcus aureus* (ATCC 6341); the Gram-negative bacteria *Escherichia coli* (ATCC 4157), *Haemophilus influenzae* (ATCC 8142), *Pseudomonas aeruginosa* (ATCC 7700), and *Proteus vulgaris* (ATCC 6896); and the fungus *Candida albicans* (ATCC 752). The bacteria were maintained at 4°C on tryptic soy agar (TSA), and *C. albicans* (ATCC 752) was maintained on potato dextrose agar (PDA) (Difco, NJ, USA) before use. *H. influenza* (ATCC 8142) was incubated in the presence of a Gas-Pac (Becton and Dickinson Co, NJ, USA) with 5% CO_2._

### Antimicrobial activity determination using the disc diffusion assay

The disc preparation was performed following the procedure outlined by the British Society for Antimicrobial Chemotherapy in their Methods for Antimicrobial Susceptibility Testing Version 7, January 2008 [15]. A single bacterial colony was removed from a streak plate of Trypticase soy agar (TSA, BD Biosciences) and incubated in Luria broth (LB, Difco) overnight with shaking at 37°C. A bacterial suspension in 2 mL of distilled H_2_O was prepared and adjusted to a density of 0.5 McFarland units, which correspond to 1.5 × 10^8^ CFU/mL. The suspension was then plated onto Mueller-Hinton agar (Difco), followed by disk deposition and incubation at 37°C for 20 hours. Growth inhibition was determined by measuring the diameter of the inhibition surrounding each disc. The relative inhibition was calculated for each extract and is represented as the percentage difference of inhibition between the positive control (antibiotic) and the extract. All of the experiments were performed in triplicate.

### Minimum inhibitory concentration (MIC) determination

The MIC is defined as the lowest concentration of antimicrobial that can inhibit the visible growth of a microorganism after overnight incubation (British Society for Antimicrobial Chemotherapy). Extracts that showed inhibition in the disc diffusion assay were further analyzed according to the procedure of Velmonte et al. with some modifications [16]. The bacterial species tested were *S. saprophyticus* (ATCC 15305), *S. aureus* (ATCC 6341), *P. aeruginosa* (ATCC 7700) and *P. vulgaris* (ATCC 6896). MIC was determined for the extracts of *Syzygium jambos, Pityrogramma calomelanos, Tapeinochilus ananassae* and *Gossypium barbadense*. Stock solutions of lyophilized aqueous extracts were serially diluted to obtain working solutions with concentrations ranging from 2.0 to 0.003 mg/mL. Wells in a sterile 96-well plate were inoculated with Luria Broth (LB) medium in the absence of bacteria (blank control sample), bacteria with medium (positive control sample), bacteria with medium and the extract dilutions (experimental samples), background control (samples consisting of medium with extract dilutions to eliminate background extract absorbance), and bacteria with Streptomycin at 10 mg/mL (negative control samples). Bacterial suspensions containing 5×10^4^ CFU/mL of *Staphylococcus aureus* (ATCC 6341), *Staphylococcus saprophyticus* (ATCC 15305), *Proteus vulgaris* (ATCC 6896) or *Pseudomonas aeruginosa* (ATCC 7700) were added to each well. The 96-well plates were incubated at 37°C for 18–20 hours. The absorbance at 625 nm was measured for each plate using a microplate reader, and background-subtracted with the background of the control (bacteria and medium). Then, the absorbance of the extracts was compared to the absorbance of the positive and negative control (Streptomycin 10 mg/mL) wells. The MIC was the concentration in the wells that showed an absorbance lower to the control well and that also gave a p-value of 0.001 or less after statistical analysis.

### Minimum bactericidal concentration (MBC) determination

The minimum bactericidal concentration (MBC) value is defined as the extract concentration at which no bacterial growth is observed in the agar nutrient plates, similar to the negative control Streptomycin (10 mg/mL). The effective concentration of the extracts in the experimental wells, as determined using the MIC assay, was used to inoculate LB plates using a sterile swab and incubated overnight at 37°C. Plates containing positive (bacteria with medium) and negative (bacteria with 10 mg/mL Streptomycin) controls for each bacterial species were also prepared. The lowest concentration of the extract at which no bacterial growth was observed was recorded as the MBC. The MBC experiments were performed in triplicate.

### Statistical analysis

The inhibition data are presented as the percentage of the positive control +/− SD. The MIC values are the concentrations of each plant that were statistically significant (value of 0.001 or less) compared with the control samples.

## Results and discussion

**Figure 1:**
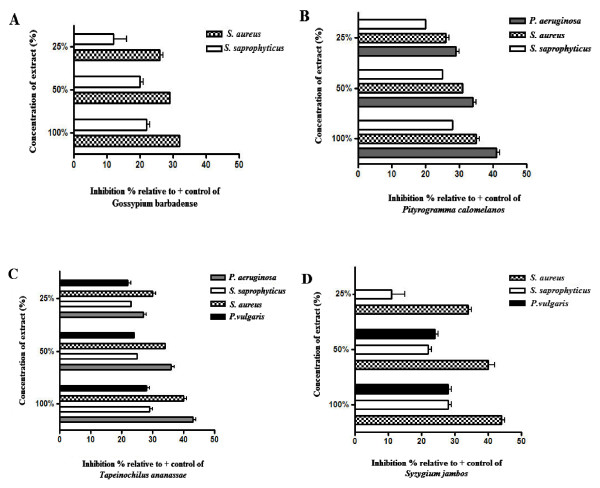
**Herbal remedies in the Caribbean that showed antimicrobial activities.** Inhibition was calculated as percentages relative to the positive control, Streptomycin- treated discs. **A**) *G. barbadense* juice; **B**) *P. calomelanos* decoction; **C**) *T. ananassae* decoction and **D**) *S. jambos* decoction.

The plant extracts listed in Table 1 were studied for their antimicrobial activities against *Staphylococcus saprophyticus* (ATCC 15305), *Staphylococcus aureus* (ATCC 6341), *Pseudomonas aeruginosa* (ATCC 7700), *Proteus vulgaris* (ATCC 6896), *Escherichia coli* (ATCC 4157), *Haemophilus influenza (ATCC 8142)*, and the fungus *Candida albicans (ATCC 752).* Of the 13 plant extracts tested, data corresponding to the extracts that showed microbial growth inhibition in the disc diffusion assays will be discussed. These extracts include *G. barbadense* fruit juice and the decoctions of *P. calomelanos*, *T. ananassae*, and *S. jambos* (Figure 1). The bacterial species inhibited by these extracts were *S. saprophyticus* (ATCC 15305), *S. aureus* (ATCC 6341), *P. aeruginosa* (ATCC 7700) and *P. vulgaris* (ATCC 6896).

Juice prepared from unripe *G. barbadense* fruit exhibited more than 25% growth inhibition of the two Gram-positive bacteria *S. aureus* (ATCC 6341) and *S. saprophyticus* (ATCC 15305) relative to Streptomycin treatment which showed 100% inhibition at 5 mg/mL. *S. aureus* (ATCC 6341) has been isolated from the discharge produced during an otitis media infection, which provides a rationale for the use of *G. barbadense* juice (juice from the unripe fruit) as an herbal remedy for this infection [17]. The antimicrobial activity was observed at a concentration of 7 mg/mL, the lowest concentration tested (Figure 1A). The MIC values that were determined in this study for *G. barbadense* against *S. aureus* (ATCC 6341) and *S. saprophyticus* (ATCC 15305) did not meet the p-value of 0.001 or less used in our analyses. This result contrasts with a study of medicinal plants from Yemen in which aqueous extracts from *G. barbadense* were not active (MIC values > 1,000 μg/mL) against *S. aureus* and *E. coli* and showed low activity (MIC = 1000 μg/mL) against *P. aeruginosa* and other bacterial strains [18]. In our study, no measureable MBC was observed for either bacterial species. Treatment for otitis media using herbal remedies has been very difficult. A study using an ethanolic extract of the commonly used herb for infectious diseases, *Echinacea purpurea* roots and seeds, did not show a decrease in the risk of otitis media infection [19]. Nevertheless, naphthofuran analogues and biologically active terpenoids from *G. barbadense* are known to exhibit growth-inhibitory and antibacterial activities [20,21]. In addition, the essential oil has been reported to show moderate activities against *S. aureus*, *E. coli* and the fungus *C. albicans*[22]. Although the antimicrobial activity in the oil might be due to the major components or to synergy between major and minor components of the oil, α-pinene, which is a terpene that comprises 12.8% of the oil, has shown efficient antimicrobial properties.

The decoction of *P. calomelanos* (at the lowest concentration of 37.5 mg/mL) inhibited the growth of *P. aeruginosa* (ATCC 7700), *S. aureus* (ATCC 6341) and *S. saprophyticus* (ATCC 15305) by 2%-20% relative to Streptomycin which showed 100% inhibition at 5 mg/mL (Figure 1B). The MIC for *P. aeruginosa* (ATCC 7700) was 2 mg/mL (p = 0.0001); however, for *S. aureus* (ATCC 6341) and *S. saprophyticus* (ATCC 15305)*,* the MIC values were not statistically significant (Table 2). None of the concentrations that were tested showed bactericidal activity. *P. calomelanos* is traditionally used to treat kidney stones. The bacterium *P. aeruginosa* has been associated with biofilm formation on kidney stones, which leads to urinary infections [23]. The use of *P. calomelanos* decoctions to treat kidney stones could be related to the growth inhibition of *P. aeruginosa*, which may justify the use of this treatment as an herbal medicine. Studies that focused on several fern families have demonstrated that ethanol and methanol extracts from these plants exhibit antibacterial properties [24,25]. Recently, a study reported the antimicrobial and modulatory antibiotic activity of the ethanol extract and the methanolic fraction from the leaves of *P. calomelanos*[26]. Neither preparation demonstrated antimicrobial activity that was clinically relevant to fungi or bacteria (MIC > 1024 μg/mL). However, in the modulation assay, both extracts modulated most of the antibiotics tested against *S. aureus*. The present study only tested aqueous extracts as a way to validate the traditional remedies employed by the surveyed population and is the first to report the antimicrobial activities of *P. calomelanos* decoctions. Phytochemical analysis of aqueous extracts of *P. calomelanos* has revealed the presence of carbohydrates and coumarin [27]. Although data regarding the specific antibiotic properties of coumarins are scarce, antimicrobial activity against bacteria, fungi and viruses has previously been reported for this class of phytochemicals [3]. Conversely, polysaccharides are commonly more effective as inhibitors of pathogen adsorption and would not be identified in the screening techniques commonly used.

**Table 2:** **MIC (p-value 0.001 or less) and MBC of extracts that showed inhibition in the disc diffusion assays**^**a**^

**Plant extracts**	**Bacteria**	**MIC (mg/mL)**	**p-value**	**MBC (mg/mL)**
*T. ananassae*	*P. aeruginosa*	0.5	0.0002	ND^b^
	*P. vulgaris*	0.5	0.0001	2.0
	*S. aureus*	0.015	0.001	ND^b^
	*S. saprophyticus*	2.0	0.0004	2.0
*P. calomelanos*	*P. aeruginosa*	2.0	0.0001	ND^b^
*S. jambos*	*S. aureus*	0.5	0.00001	1.0
	*S. saprophyticus*	0.5	0.001	2.0
	*P. vulgaris*	0.031	0.001	1.0

a) All the extracts that showed inhibition in the disc diffusion assay were analyzed against the bacteria isolates *S. saprophyticus* (ATCC 15305), *S. aureus* (ATCC 6341), *P. aeruginosa* (ATCC 7700) and *P. vulgaris* (ATCC 6896).

b) *ND*, Not detected.

The decoction of *T. ananassae* at 15.3 mg/mL, the lowest concentration tested, inhibited the growth of two Gram-positive and two Gram-negative bacterial strains by 22%-30% compared to the 100% inhibition of Streptomycin at 5 mg/mL (Figure 1C). Specifically, the growth inhibition values of *P. aeruginosa* (ATCC 7700), *S. saprophyticus* (ATCC 15305), *S. aureus* (ATCC 6341) and *P. vulgaris* (ATCC 6896) were 29%, 22%, 32% and 24%, respectively. The MIC values for *T. ananassae* were 0.5 mg/mL for *P. aeruginosa* (ATCC 7700), and *P. vulgaris* (ATCC 6896) (p = 0.0002 and 0.0001, respectively), 0.015 mg/mL for *S. aureus* (ATCC 6341) (p = 0.001) and 2.0 mg/mL for *S. saprophyticus* (ATCC 15305) (p = 0.0004) (Table 2). *T. ananassae* displayed bactericidal activity against *P. vulgaris* (ATCC 6896) and *S. saprophyticus* (ATCC 15305) at concentrations of 2.0 mg/mL for both species (Table 2). Our study showed that T*. ananassae* inhibits the growth of pathogens associated with opportunistic infections of burns and skin infections such as *P. aeruginosa* and *S. aureus*, respectively [28]. *P. aeruginosa* has become an important cause of infection, especially in immunocompromised patients, such as diabetic and cancer patients [29]. The antibiotic activities of *T. ananassae* are important because people with diabetes are vulnerable to infections as a result of low blood flow to the extremities [30] and because of certain types of neuropathy that cause dry and cracked skin. Additional antimicrobial activity has been reported for *T. ananassae* against the Gram-negative bacterium *Helicobacter pylori*[31].

Decoctions of *Syzygium jambos* leaves inhibited *S. aureus* (ATCC 6341) and *S. saprophyticus* (ATCC 15305) by 34% and 11%, respectively, at the lowest concentration studied (10.5 μg/μL) relative to Streptomycin which showed 100% inhibition at 5 mg/mL. *S. jambos* at 20.9 mg/mL also inhibited the Gram-negative *P. vulgaris* (ATCC 6896) by 24% relative to Streptomycin which showed 100% inhibition at 5 mg/mL (Figure 1D). The MIC value for *S. aureus* (ATCC 6341) and *S. saprophyticus* (ATCC 15305) were 0.5 mg/mL (p = 0.00001 and 0.001, respectively), and the MIC value for *P. vulgaris* (ATCC 6896) was 0.031 mg/mL (p = 0.001) (Table 2). *S. jambos* was the only extract that showed bactericidal activity against all of the isolates that were tested. The MBC value for both *S. aureus* (ATCC 6341) and *P. vulgaris* (ATCC 6896) was 1.0 mg/mL, and the MBC value for *S. saprophyticus* (ATCC 15305) was 2.0 mg/mL (Table 2). Previously, Lin et al. reported that aqueous and acetone extracts from the bark of *Syzygium jambos* had activity against several species of *S. aureus* with MIC values that ranged from 0.50 to 0.75 mg/mL and suggested that more active inhibitory compounds are found in the leaves (0.21 to 0.83 mg/mL) than in the bark [32]. Additionally, methanolic extracts of *Syzygium aromaticum* showed inhibition against *S. aureus, Staphylococcus epidermidis, Streptococcus pyogenes, Salmonella enterica serovar Typhi* and *P. aeruginosa* with MICs ranging from 31.25-250 μg/mL [33]. We did not find antimicrobial activity against *P. aeruginosa*, which suggests that variation in the polarities of the extraction solvents and the preparation methods used to make the extracts that were studied may account for the difference in the activities of these two species.

For nearly all of the plant species that have significant uses but that did not show antimicrobial activity, there is evidence that their activity varies significantly with the polarity and the concentration of the extracts, which in turn determines the phytochemicals responsible for the antibiotic effects [3,34]. For instance, the antimicrobial effects of hydroalcoholic extracts from the leaves of *Plectranthus amboinicus* have been reported against *S. aureus* with a MIC of 9.3 mg/mL [35]. In another study [36], seven out of ten *S. aureus* strains obtained from exudates of otitis externa were sensitive to *P. amboinicus* extracts at concentrations ranging from 1.25 to 5.0 mg/mL. In particular, the essential oil of *P. amboinicus* was active against two of the strains tested at 4% and 8% (w/V). This result is noteworthy because the decoction, which corresponds to the traditional preparation that is used, significantly resembles the fraction obtained from a steam distillation, which carries the essential oils that would have been inactive in solution at the concentrations obtained in this study (5.3 – 21.3 μg/μL). The authors do not report the polarity of the extracts, although the use of hydroalcoholic extracts was suggested.

The polar methanolic extracts from the leaves and bark of *Aristolochia trilobata* showed activity against *E. coli* with a MIC value of 2.5 mg/mL [37]. This MIC value is comparable to the concentration of the decoctions tested in this study, which were 3.22 and 3.53 mg/mL for the stem and leaves, respectively. It is known that methanol extractions are more exhaustive than those of the aqueous or less polar organic and non-polar organic solvents. Therefore, the compounds that are responsible for the observed activity could be absent from the aqueous decoctions. However, the authors previously reported that the traditional use of this plant species has to be discouraged due to the mutagenic and possibly human carcinogenic properties of the aristolochic acids present in the methanol extracts and possibly in the water preparations of aerial parts of the plant.

Serrulatane quinonoid biflorin, which is isolated from the root tissue of *Capraria biflora*, has been reported to exhibit antimicrobial activities against Gram-positive bacteria [38]. This activity is consistent with the traditional use to treat conjunctivitis, which may be associated with infections by *S. aureus*, *S. epidermidis* and *Propionibacterium spp.*, among others. Thus far, the activity of aqueous extracts from the leaves has not been reported.

Aqueous extracts from the rhizomes of *Costus speciosus* have been reported to exhibit antimicrobial activity against *S. aureus* at a concentration of 200 mg/mL [39]. The report describes the traditional system of administration studied herein, but given that the concentrations tested in our study varied from 11.8 – 47.3 μg/μL, a continuous administration of the decoction would be required to explain the traditional use for kidney infections.

Although there are several reports of the broad spectrum antimicrobial activity of *Cucurbita moschata* oil and seed extracts, no reports were found on the activity of decoctions from the leaves [40]. Likewise, only ethanol and acetone:ethyl acetate (1:1) extracts of the aerial parts of *H. rosa-sinensis* have been studied previously [41,42]. The reported MIC and MBC values for the ethanolic extract of the flowers against *S. aureus* were 20 mg/mL. Thus, although the activities reported indicate that antimicrobial compounds are present in more polar solvents, higher concentrations than that found in the traditional remedy are needed to assess the antibacterial activity.

The results for *Plantago major* are similar to those described above. That is, at a concentration of 2 mg/mL in the hydroalcoholic extract, which is higher than those found in the decoctions (7.2 – 28.7 μg/μL), the authors reported MIC values of 1 mg/mL for *S. aureus* and > 1 mg/mL against *E. coli, P. aeruginosa*, *C. albicans* and others [43]. Furthermore, the antibacterial properties of lyophilized and fresh water extracts of *Abelmoschus esculentus* were effective against all bacterial strains tested, including *S. aureus*, *E. coli* and *P. aeruginosa*. The fresh extract displayed better antibacterial properties than the lyophilized extract [44]. The MIC values for peeled and unpeeled *A. esculentus* were 6.4 and 12.8 mg/mL for *S. aureus* and *P. aeruginosa*, respectively. In this case, the lipid fraction of *A. esculentus* was found to be responsible for the antibacterial properties, whereas the protein and polysaccharide fractions displayed no antimicrobial activity. The concentration of the *A. esculentus* decoction that was prepared in this study according to the traditional use reported during the ethnopharmacological survey was 12.6 mg/mL.

This study did not find antimicrobial activity that validates the traditional use of several herbal decoctions that showed significant use during TRAMIL ethnopharmacological surveys. Two separate analyses, the disc diffusion and the MIC assays, demonstrated that fewer than 33% of the herbal remedies that were examined exhibited antibacterial activities. This result is not discouraging because several factors, in addition to those studied, could determine the effectiveness of the extracts. First, the extracts could contribute to the treatment of disease, not necessarily by exerting an antibacterial response but by promoting an anti-inflammatory response. This effect was shown by Yang and collaborators, who demonstrated the antimicrobial and anti-inflammatory properties of the essential oils from *Citrus sunki*[45]. In addition, aqueous extracts are typically less potent formulations than those prepared with organic solvents such as methanol [46]. The solvent and the extraction system may modify the final results as described in the study of *Argemone mexicana*[47]. In this study, although the various solvent extracts examined were effective, the methanolic extracts showed maximum inhibition against the tested microorganisms, followed by hot aqueous extracts and cold aqueous extracts.

## Conclusions

This study examined the inhibitory activity of Caribbean herbal remedies, which are treatments that are generally used by the surveyed populations for otitis media, kidney stones, skin burns, fever and conjunctivitis, which are associated with infections caused by Gram-positive and Gram-negative bacteria. Considering that antimicrobials are typically regarded as bactericidal if the MBC is nearly four times the MIC, this study identified the novel bactericidal properties of *T. ananassae* and *S. jambos* decoctions, which are commonly used as diabetes adjuvants in Puerto Rico. The bactericidal activities of these preparations will be extremely useful when treating patients with compromised immune systems, such as diabetes patients. Additionally, this article is the first report of the antimicrobial activities of *G. barbadense* and *P. calomelanos* decoctions. The findings that are reported here, which are the result of a screen for the antimicrobial activities of traditional Caribbean herbal remedies, will be presented to the TRAMIL program to inform future TRADIF activities.

## Competing interests

The authors state no conflict or competing interests are associated with the present study.

## Authors’ contributions

CLM and JGS designed the study and contributed to the preparation of the manuscript and data analysis. CLM carried out the inhibition assays. JGS collected plants for the study. IB supplied the results of ethnopharmacological surveys. All the authors approved and reviewed the final draft of the manuscript.

## Pre-publication history

The pre-publication history for this paper can be accessed here:

http://www.biomedcentral.com/1472-6882/13/126/prepub
